# Road traffic noise and hypertension: results from a cross-sectional public health survey in southern Sweden

**DOI:** 10.1186/1476-069X-8-38

**Published:** 2009-09-10

**Authors:** Theo Bodin, Maria Albin, Jonas Ardö, Emilie Stroh, Per-Olof Östergren, Jonas Björk

**Affiliations:** 1 Department of Occupational and Environmental Medicine, Lund University Hospital, Lund, Sweden; 2 Department of Physical Geography and Ecosystems Analysis, Lund University, Lund, Sweden; 3 Department of Clincal Sciences, Malmö, Division of Social Medicine and Global Health, Malmö University Hospital, Malmö, Sweden; 4 Competence Center for Clinical Research, Lund University Hospital, SE-221 85 Lund, Sweden

## Abstract

**Background:**

Results from studies of road traffic noise and hypertension are heterogeneous with respect to effect size, effects among males and females and with respect to effects across age groups. Our objective was to further explore these associations.

**Methods:**

The study used cross-sectional public health survey data from southern Sweden, including 24,238 adults (18 - 80 years old). We used a geographic information system (GIS) to assess the average road noise (LAeq 24 hr) at the current residential address. Effects on self-reported hypertension were estimated by logistic regression with adjustment for age, sex, BMI, alcohol intake, exercise, education, smoking and socioeconomic status.

**Results:**

Modest exposure effects (OR ≈ 1.1) were generally noted in intermediate exposure categories (45 -64 dB(A)), and with no obvious trend. The effect was more pronounced at > 64 dB(A) (OR 1.45, 95% CI 1.04 - 2.02). Age modified the relative effect (p = 0.018). An effect was seen among middle-aged (40 - 59 years old) at noise levels 60 - 64 dB(A) (OR = 1.27, 95% CI 1.02 - 1.58)) and at > 64 dB(A) (OR = 1.91, 95% CI 1.19 - 3.06)). An effect was also indicated among younger adults but not among elderly. No apparent effect modification by gender, country of origin, disturbed sleep or strained economy was noted.

**Conclusion:**

The study supports an association between road traffic noise at high average levels and self-reported hypertension in middle-aged. Future studies should use age group -specific relative effect models to account for differences in prevalence.

## Background

Road traffic is the most important source of community noise. Even though very high levels of traffic noise, i.e. average day-night A-weighted equivalent noise level exceeding 65dB(A), seem to have stabilized in some countries, the group living in dwellings exposed to 55-65 dB(A) is increasing[1] In total approximately 30% of the population in the European Union still is exposed to a day-night average of traffic noise exceeding 55dB(A)[2]

Non-auditory physical health effects that are biologically plausible in relation to noise exposure and annoyance from noise exposure include changes in blood pressure, heart rate, and levels of stress hormones[3] The biological mechanism linking noise to hypertension is thought to be mediated through sympathetic and endocrine stress response with subsequent acute changes in vascular tension. The hypothesis is that long-time exposure to noise could result in lasting cardiovascular changes such as atherosclerosis, and increase cardiovascular risk as well as hypertension. [4-6]

Effects of road and air traffic noise on ischaemic heart disease, mean blood pressure and hypertension were reviewed in 2006[7] No apparent indications of higher risk for ischaemic heart disease (including myocardial infarction) were found at average daytime levels below 60 dB(A), but increased risks were relatively consistently found at higher average levels, which has also been confirmed in a recent meta-analysis. [8] Traffic noise seemed to moderately increase mean blood pressure in children, whereas the findings in adults were inconsistent. With respect to traffic noise and hypertension, the review showed a heterogeneous picture. A consistent positive association between aircraft noise and hypertension was found, with growing evidence since the previous review in this area[9] A number of recent studies (after the review in 2006) have provided further evidence for associations between traffic noise and ischaemic heart disease, [10] between aircraft noise and hypertension, [11,12] and between road traffic noise and hypertension. [13-16] However, also recent studies of road traffic noise and hypertension are heterogeneous with respect to effect size, [10,16] effects among males and females [13,14] and with respect to effects across age groups. [15] One plausible explanation for the diverging results is that the overall association between road traffic noise and hypertension is weak at average noise levels typically found in most urban environments. It is also conceivable, however, that effects are more marked at higher exposure levels, in certain age groups or in other subgroups of the population, but investigating such associations generally requires large population studies. In a large pooled European population sample based on blood pressure measurements, the prevalence of hypertension increased from 27% in the age group 35 - 44 years to 78% among subjects 65 - 74 years old[17] Given this dramatic increase in the prevalence of hypertension with age, it is most unlikely that the relative effect of road noise exposure is constant across age groups, but few studies have used exposure - disease models that allow for effect modification by age[15]

The aim of this study was to further explore the association between road traffic noise and hypertension, and to investigate whether this association was differential with respect to gender or age, or especially marked in vulnerable groups within the population. The study used cross-sectional data from a large public health survey in southern Sweden, combined with road traffic noise data assessed for the current residential addresses of the survey participants using a geographical information system (GIS).

## Methods

### Study subjects

The study, which was conducted in accordance with Swedish law of ethics, was based on data from an extensive public health survey (130 questions) in the Scania region in southern Sweden[18] All persons 18 - 80 years old, living in this region on 30 June 2004, constituted the study population (N = 855,599). The population was stratified by gender and geographical area, resulting in 2 * 62 = 124 different strata. Samples were randomly selected from the population registry such that an approximately equal number of individuals were contacted in each stratum. In total, the questionnaire was sent by mail to 46,200 persons, while 2,800 were randomly selected to answer the questionnaire by telephone interview.

The questionnaire consisted of detailed questions regarding self-reported illness, health and well-being, life-style habits such as smoking, alcohol consumption, physical exercise and diet, social relations, treatment with drugs, healthcare use, occupation and work environment, financial situation, educational level, ethnic background and residential environment.

### Assessment of hypertension and selection of confounders

A subject was defined as hypertensive if an affirmative answer was given to any of the following two survey questions: 1) "Do you have the following health problem /.../ Hypertension?", 2) "Have you, during the last three months, used any drug or preparation against hypertension". The overall proportion of coherent answers between the two questions was 93%. The coherence was lower among younger respondents and among men. We investigated confounding from variables a priori considered to be possible risk factors for hypertension: [19] sex, age, BMI, exercise, education, alcohol consumption, smoking and socioeconomic status. Smoking was included even though there is some controversy whether it leads to hypertension or not. [20] The confounders were either continuous or categorized, as presented in Table 1.

**Table 1:** The distribution of confounders between "cases" i.e. individuals reporting hypertension and "non-cases" being those not reporting hypertension divided in three groups by level of road traffic noise exposure.

	Cases	Non-cases		
LAeq 24 hr (dB)	All (0-71)	<45	45-54	≥55
Individuals per group	4644	4688	9337	5569
				
	median (q1-q3)	median (q1-q3)	median (q1-q3)	median (q1-q3)
				
Age	61 (53-69)	45 (35-57)	45 (33-58)	42 (30-57)
BMI	26.7 (24.3-29.4)	24.4 (22.3-26.9)	24.5 (22.2-27.1)	24.2 (22-26.6)
Alcohol consumption index	3 (1-4)	3 (2-4)	3 (2-4)	3 (2-5)
				
	N (%)	N (%)	N (%)	N (%)
				
Smoking				
Never	2319 (49.9)	2763 (58.9)	5197 (55.7)	2992 (53.7)
Former	1485 (32)	1087 (23.2)	2180 (23.3)	1258 (22.6)
Current (not every day)	159 (3.4)	187 (4)	463 (5)	305 (5.5)
Current (every day)	681 (14.7)	651 (13.9)	1497 (16)	1014 (18.2)
Sex				
Man	2439 (52.5)	2101 (44.8)	4115 (44.1)	2447 (43.9)
Woman	2205 (47.5)	2587 (55.2)	5222 (55.9)	3122 (56.1)
Education				
Lower education	3502(75.4)	3054 (65.1)	6004 (64.3)	3405 (61.1)
Higher education	1142(24.6)	1634 (34.9)	3333 (35.7)	2164 (38.9)
Exercise				
Mainly sedentary	819 (17.6)	551 (11.8)	1165 (12.5)	741 (13.3)
Light/Moderate	3306 (71.2)	3366 (71.8)	6561 (70.3)	3823 (68.6)
Regular	519 (11.2)	771 (16.4)	1611 (17.3)	1005 (18)
Socioeconomic status				
High level non-manual	291 (6.3)	545 (11.6)	1030 (11)	664 (11.9)
Middle level non-manual	427 (9.2)	836 (17.8)	1549 (16.6)	839 (15.1)
low level non-manual	282 (6.1)	427 (9.1)	761 (8.2)	429 (7.7)
Skilled manual	349 (7.5)	537 (11.5)	1083 (11.6)	564 (10.1)
Unskilled manual	479 (10.3)	690 (14.7)	1406 (15.1)	799 (14.3)
Self-employed/Farmer	205 (4.4)	407 (8.7)	465 (5)	262 (4.7)
Disabilty pension	424 (9.1)	176 (3.8)	377 (4)	231 (4.1)
Unemployed	175 (3.8)	190 (4.1)	431 (4.6)	354 (6.4)
Stuadent	77 (1.7)	296 (6.3)	829 (8.9)	628 (11.3)
Old-age pension	1833 (39.5)	514 (11)	1252 (13.4)	718 (12.9)
Long-term sickness absence	102 (2.2)	70 (1.5)	154 (1.6)	81 (1.5)

### Assessment of road traffic noise

No measurements of noise levels were conducted. Instead, we used a geographic information system (GIS) to assess the outdoor noise exposure from traffic. Current residential addresses for the participants in the public health survey and road traffic data were geocoded. Road traffic data included 21,397 road segments (17,339 administrated by the Swedish Road Administration, and 4,058 by local municipalities). The number of vehicles was available for 82% of the road segments. Speed limits were available for >95% of the segments. For road segments without traffic data, mean values were assigned to each segment on the basis of existing data for the included road types[21] Using the road traffic data, we used a simplified version of the Nordic prediction method for road traffic noise [see the reports by Lyse Nielsen [22] and Jonasson et al [23] for a complete description] to estimate noise exposure for the residential locations of the study participants. In short, the Nordic prediction method first calculates the unattenuated noise level 10 meters from the road center using the number of light and heavy vehicles and the speed limit of each road segment. Corrections were then calculated for (i) the distance between the source (the road) and receptor, for which the noise levels decrease by 3 dB(A) with a doubling of the distance, (ii) attenuation due to ground surface type and noise barriers [the attenuation of noise depends on surface type with less attenuation for hard surfaces (asphalt, water, concrete) and more attenuation for soft surfaces (vegetation, grass, etc)], and (iii) additional corrections for special cases (including very steep topography, reflection from buildings, etc).

In this study, we had to simplify the Nordic prediction method by using corrections for distance and surface type only. We were not able to correct for noise barriers and the additional special cases already mentioned, as no such data was available. We assumed flat ground in all cases and soft surfaces between the residence and the road for the participants living in the countryside, while a hard surface was assumed for the participants living in more densely populated areas. We had no data indicating the floor of the apartment building on which the residences were located, and we therefore estimated the noise level on the ground floor for all of the residences.

We estimated the A-weighted equivalent noise level over a full day (24 hours, LAeq 24 hr) in dB(A). Estimated noise levels during the day and night were too strongly correlated with the noise level during a full day to be used for separate analyses. Using the number of vehicles (light and heavy) and the speed limit for each road segment, we calculated LAeq 24 hr for each 25-meter zone up to 500 meters from the center of the road. As subjects may appear in noise zones for more than one road segment, the maximum values for LAeq 24 hr across all of the road segments near the residence were extracted for each person and used for further analyses. Hence exposure refers to the most exposed façade of the residence.

Estimated road noise levels were compared to reported annoyance using the survey question "Are you disturbed by road traffic noise in your home?" The response alternatives were "every day"; "several times per week"; "more seldom" and "Never".

### Statistical analysis

Standard statistical methods were applied using SPSS 16.0 for windows (SPSS Inc, Chicago IL, USA). We used logistic regression with hypertension as outcome variable (defined by the two survey questions given above) with average road noise exposure during a full day (LAeq 24 hr) either entered as a continuous or as a categorical variable in 5 dB(A)-intervals. The highest noise levels, ranging from 65 to 71 dB(A), were merged into one group (>64). Reference category for both the continuous and the categorical exposure variable was all subjects with average road noise exposure below 45 dB(A). Three different types of logistic regression models were analyzed. The first model was unadjusted, the second partly adjusted model included sex, age and BMI as covariates, while the third fully adjusted model also included exercise, education, alcohol consumption, smoking and socioeconomic status. Effect estimates were presented as odds ratios (ORs) with 95% confidence intervals (CIs). Departure from a common, fully adjusted, relative effect model in important subgroups was investigated by adding a multiplicative interactive term (a × b) based on road noise exposure (a; continuous) and the investigated subgroup variable (b; categorical). The investigated subgroups were defined according to sex, age, years in residence, country of birth (Sweden or abroad), strained economy (Q: Have you experienced difficulties paying bills on time the last 12 months; "every month" and "approximately half of the months" vs "once or twice" and "never") and disturbed sleep (Does traffic noise cause difficulties sleeping, falling asleep or resting due to traffic noise "at least once a day" and "at least once a week" vs "more seldom" and "No, Never"). The p-value of the interaction term was reported; p-values near or below 0.05 were interpreted as signs of departure from a common relative effect model. The prevalence of hypertension varied extensively across subgroups and subgroup-specific effect estimates were therefore obtained by fitting a fully adjusted logistic regression model to each subgroup separately.

## Results

**Figure 1:**
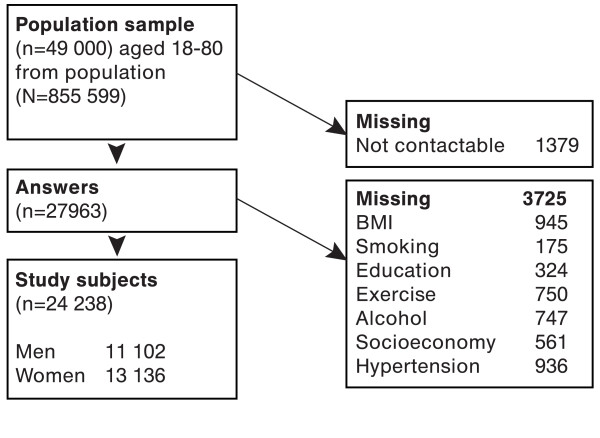
**Flowchart describing study population reduction through the different steps of inclusion and exclusion**.

Answers were obtained from 27,963 of persons after three reminder letters (59% participation rate). The participation rate was higher among females, elderly, persons born in Sweden, and among persons with high education and income. All participants did not accurately complete all questions. Complete data on hypertension and all relevant confounders (see next section) were obtained for 24,238 persons (11,102 men and 13,136 women; Figure 1).

**Figure 2:**
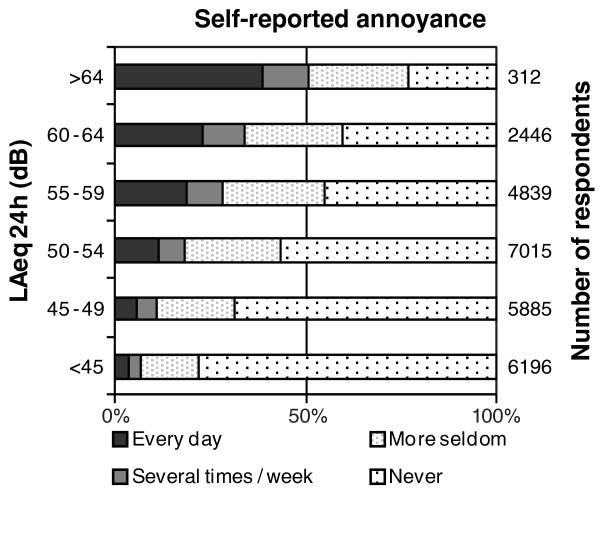
**Bars showing proportion and level of self-reported disturbance at home due to road traffic noise (x-axis) at different levels of noise exposure in our model (y-axis)**.

Analysis of correlation between our noise-exposure model (LAeq 24 h dB(A)) and selfreported annoyance was carried out. A clear pattern with increasing annoyance at increasing road noise levels was found. (Figure 2; n = 26,693 study subjects answered the question).

Table 1 shows the distribution for covariates and various risk factors of hypertension, stratified by case status (hypertensive vs. non-hypertensive according to the survey). Clear differences between cases and non-cases were noted for most variables. Non-cases were further stratified by average level of road traffic noise exposure: <45 dB(A), 45-54 dB(A) and ≥55 dB(A). The median age was three years younger in the non-case group with highest exposure compared to the two other non-case groups. Road noise exposure among the non-cases was also related to current smoking, education, and socioeconomic status (farmers underrepresented and students overrepresented in the highest exposure category). Unadjusted analyses were therefore likely to be confounded to some extent by age and possibly also by other factors. Modest exposure effects (OR ≈ 1.1) were generally noted for the four intermediate exposure categories (45-49, 50-54, 55-59, 60-64 dB(A)). No obvious trend was found between the categories (Table 2). The effect was more pronounced in the highest exposure category > 64 dB(A); OR(95%CI) 1.52 (1.09-2.11) when adjusting for age, sex and BMI.

**Table 2:** OR (CI 95%) for hypertension associated to different levels of road traffic noise exposure using three different models of adjusting.

Laeq 24 h (dB)	N*	Median age	Hypertension prevalence	Unadjusted OR (CI 95%)	Age, BMI, Sex OR (CI 95%)	Fully adjusted OR (CI 95%)
<45	5707	48	17.90%			
						
45-49	5362	50	20.40%	1.18 (1.07-1.29)	1.10 (0.99-1.22)	1.09 (0.99-1.21)
50-54	6290	49	19.40%	1.11 (1.01-1.22)	1.08 (0.98-1.20)	1.08 (0.97-1.19)
55-59	4395	47	19.20%	1.10 (0.99-1.21)	1.11 (0.99-1.24)	1.10 (0.98-1.23)
60-64	2202	45	18.40%	1.04 (0.92-1.18)	1.14 (0.99-1.31)	1.12 (0.97-1.29)
>64	282	45	20.90%	1.22 (0.91-1.63)	1.52 (1.09-2.11)	1.45 (1.04-2.02)
5dB(A) increase	24238			1.00 (0.98-1.03)	1.03 (1.00-1.07)	1.03 (1.00-1.06)
10dB(A) increase	24238			1.01 (0.95-1.06)	1.07 (1.01-1.14)	1.06 (1.00-1.13)

* N is the same for all three models, adjusted as well as unadjusted, e. i. uses the same individuals

**Figure 3:**
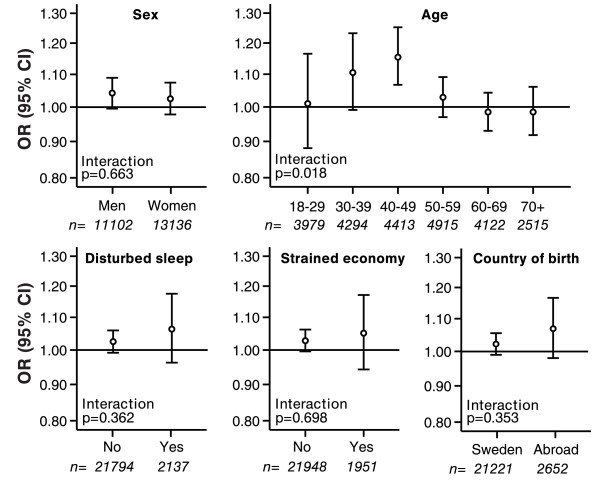
**Fully adjusted odds ratios and 95% confidence intervals of hypertension associated to a estimated 5 dB(A) increase in traffic noise by age, sex, country of birth, sleeping and resting problems due to traffic noise, difficulties paying bills and number of years living in the same residence**. The subgroup-specific effect estimates were obtained by fitting a fully adjusted (see Methods) logistic regression model to each subgroup separately.

Departure from a common relative effect model was noted for age (p for interaction = 0.018; Figure 3). An exposure effect of road traffic noise was indicated in the youngest age group (18 - 39 years old) at exposure levels 60 - 64 dB(A) OR(95%CI) 1.47 (1.01-2.14), whereas the estimated effect at higher exposure levels was imprecise (Table 3). Among middle-aged (40 - 59 years old), effects of road noise exposures were seen in the 60-64 and >64 dB(A) categories. OR(95%CI) 1.30 (1.05-1.61) 2.03 (1.28-3.24) respectively when adjusted for age, sex and BMI. A finer stratification of age indicated that significant exposure effects were present only in the age span 30 - 49 years old (Figure 3). There was no clear association between road traffic noise and prevalence of hypertension in the oldest age group (60 - 80 years old), but the effect estimate for the highest exposure category (> 64 dB(A)) was again imprecise OR(95%CI) 1.10 (0.64-1.89). Effect modification was also indicated for years in residence, with a similar, bell-shaped pattern as for age (p for interaction = 0.054; not in figures). However, age and years in residence were interrelated and the effect modification by years in residence did not remain (p = 0.29) when adjustment for effect modification by age was included in the same model. No apparent difference in effect between the sexes was discerned (Figure 3). The effect was not markedly different in subjects born abroad, in subjects with disturbed sleep, or with strained economy, but here the confidence intervals were all wide.

**Table 3:** OR and 95% CI of hypertension associated with different levels of road traffic noise exposure (categorical) in three different age groups using two models of adjusting.

Age	Laeq 24 h (dB)	N = *	Prevalence	Age Sex, BMI OR (95% CI)	Fully adjusted OR (95% CI)
18-39	<45	1782	4.0%		
	45-49	1637	4.8%	1.17 (0.84-1.62)	1.16 (0.83-1.61)
	50-54	2190	4.6%	1.19 (0.88-1.63)	1.17 (0.85-1.60)
	55-59	1638	4.3%	1.12 (0.80-1.57)	1.10 (0.78-1.55)
	60-64	906	5.5%	1.47 (1.01-2.14)	1.44 (0.99-2.10)
	>64	120	5.0%	1.38 (0.58-3.26)	1.30 (0.55-3.09)
	5dB(A) increase	8273		1.07 (0.98-1.16)	1.06 (0.97-1.16)
	10dB(A) increase	8273		1.14 (0.96-1.35)	1.13 (0.95-1.34)
					
40-59	<45	2481	16.7%		
	45-49	2189	17.0%	1.00 (0.85-1.17)	0.99 (0.84-1.17)
	50-54	2272	17.3%	1.01 (0.86-1.18)	1.00 (0.85-1.17)
	55-59	1531	18.3%	1.10 (0.93-1.32)	1.08 (0.91-1.29)
	60-64	753	21.1%	1.30 (1.05-1.61)	1.27 (1.02-1.58)
	>64	102	28.4%	2.03 (1.28-3.24)	1.91 (1.19-3.06)
	5dB(A) increase	9328		1.08 (1.03-1.13)	1.07 (1.02-1.12)
	10dB(A) increase	9328		1.16 (1.06-1.28)	1.15 (1.04-1.26)
					
60-80	<45	1444	36.8%		
	45-49	1536	41.7%	1.19 (1.03-1.39)	1.19 (1.02-1.38)
	50-54	1828	39.8%	1.14 (0.99-1.32)	1.13 (0.98-1.32)
	55-59	1226	40.4%	1.14 (0.97-1.33)	1.12 (0.95-1.32)
	60-64	543	36.3%	0.94 (0.76-1.16)	0.92 (0.74-1.14)
	>64	60	40.0%	1.10 (0.64-1.89)	1.05 (0.61-1.82)
	5dB(A) increase	6637		0.99 (0.95-1.04)	0.99 (0.94-1.03)
	10dB(A) increase	6637		0.98 (0.90-1.07)	0.97 (0.89-1.06)

* N is the same for both adjusted models, e. i. uses the same individuals

Additional OR and 95% CI of hypertension associated to a 5 and 10 dB(A) increase in noise exposure (continuous)

All analysis used the same individuals regardless of adjustment. Since there was a population reduction for each added confounder except age and sex, an additional analysis including all subjects with available data on hypertension, age, sex and BMI (n = 26,059) was done that did not apparently differ from the original analysis (Table 2). In the model adjusting for these three confounders, OR (95% CI) for hypertension in the 60-64 dB(A) and >64 dB(A) groups were 1.15 (1.01-1.31) and 1.51 (1.11-2.07) respectively.

## Discussion

Modest exposure effects were generally noted in all age groups at average road noise levels below 60 dB(A). More marked effects were seen at higher exposure levels among relatively young and middle-aged, whereas no effects at higher levels were discerned in the oldest age group (60 - 80 years old). Few subjects had average exposure levels >64 dB(A) and our effect estimates at such levels were therefore imprecise. No difference in effect between the sexes could be detected.

Major strengths of our study were the large number of participants and the extensiveness of the questionnaire, which made detailed confounding control possible. The information to respondents consisted of general information about the purpose of the public health survey, i.e., collection of health-related data in order to improve health-care planning and prevention, nowhere stating that road traffic noise and hypertension specifically would be studied. A broad questionnaire with a wide scope may thus decrease selection and information bias in a study with a specific aim as ours. However, there was a substantial proportion of non-responders. The participation rate was 59% and differential with respect to socioeconomic factors[18] Selection bias of the effect estimates would occur if the association between road noise exposure and participation differed among hypertension cases and non-cases. Socioeconomic status was positively associated with hypertension but not strongly related to road noise exposure in our study. It is thus likely that selective participation in our study contributed to an underestimation of the prevalence of hypertension at the population level, but it is not obvious that road noise exposure was strongly associated with participation.

Information bias must also be considered. Our definition of hypertension may have many implications. We did not measure the blood pressure ourselves and rely solely on self-reporting. A recent study concluded that as many as two-thirds of the hypertension cases were missed using self-reporting,[24] although other studies have shown sensitivity of 71%[25] However, self-reported doctor diagnosis of hypertension has been shown to have high specificity (96.4% and 91% respectively in the two studies). The first study also showed that sensitivity varied between different social groups and ages. Many patients may also go undiagnosed. Even though there are well-known definitions of hypertension used in Sweden, one could expect that the actual diagnostic threshold varies between physicians and over time. A study from 2003 reported hypertension prevalence in Sweden being approximately twice as high as in our study[17] In a recent study from the Netherlands, somewhat lower effect estimates associated with road noise exposure were seen when self-reported antihypertensive treatment were used rather than actual blood pressure measurements and pharmacy reports[15]

Our assessment of road noise exposure was detailed and based on actual data on traffic intensity for a majority of the road segments. Data on vehicles for road segments belonging to the local municipalities were included, which is important especially for those who live in an urban environment. A limitation was that we were only able to separate between urban (hard surface) and rural (soft surface) areas. We did not have data on noise barriers, window glassing and floor level which is of interest, especially in urban areas. Preliminary results from an ongoing study in Scania's largest urban area (Malmö) show that the simplified Nordic prediction model only marginally overestimates the exposure compared to a gold-standard model. The median difference was +1dB(A); Quartiles: -3, 7 dB(A); 2.5-97.5 percentiles: -10, 18 dB(A) (n = 2,966) with a slight trend towards larger over-estimations at higher noise levels. [26] The precision error is of classical type. [27] All above mentioned flaws in the simplified model lead to an underestimation of our results and might have implications on lower noise levels were the relation to hypertension was not significant. Reassuringly, effects on the categorical analysis where our main findings lay should be marginal, whereas the continuous analysis might suffer from the low precision. On the other hand side, we observed a clear correlation between modelled exposure and self-reported annoyance from road traffic noise, indicating a reasonable ranking of current exposure across study subjects.

We only had data on the current residential address, which means that the exposure assessment does not necessarily reflect long-term exposure. However, most subjects (66%) had lived at least five years at the current address. Average road noise exposure < 45 dB(A) was used as reference category. The prevalence of annoyance (defined as annoyed every day or several times/week was 5% at 30 - 34 dB(A), 6% at 35 - 39 dB(A) group and 8% at 40 - 44 dB(A) group, indicating that the cut-off level could have been another, lower one. However we chose < 45 dB(A) considering the fact that other recent studies have used this as reference-point making it easier to compare our results. This also reduced the risk of misclassification among those living outside the modelled 500-meter noise zone, which were all assessed as being below 45dB(A).

The survey included the question "How often does noise occur in your work so that you have to raise your voice in a conversation?" as an attempt to explore noise exposure at work. Four alternative answers were available; "Every day"; "Some days a week"; "More seldom" and "Never". Noise at work is a known risk factor but was not included in the analysis since no association with hypertension was seen in our study (not in results). The lack of association might be explained by the formulation of the question regarding noise work which, most likely, was not specific enough. We did not adjust for air pollution. Pollution is clearly associated with cardiovascular death [28] and has experimentally been found to cause hypertension,[29] however, to the best of our knowledge, there is no convincing epidemiological evidence that air pollution is associated with hypertension. Marginal changes in overall associations were seen in the recent study from the Netherlands when adjustments for particular matters (PM10) were conducted[15]

The overall association between hypertension and road traffic noise is within the span of other recent findings. (Table 4) However, there are some notable differences. The prevalence of hypertension increases dramatically with age and a common relative effect model for the entire target population (18 - 80 years old in our study), regardless whether the (self-reported) prevalence is e.g. 4% or 40%, does therefore not seem plausible. Surprisingly few studies have used separate effect models for different age groups. Our finding, with an exposure effect limited to relatively young and middle-aged, is fairly consistent with the recent study from the Netherlands, although the grouping of age differs between the two studies[15]

**Table 4:** Estimated effects of a 10 dB LAeq 24 hr increase in road traffic noise exposure on risk for hypertension in recent studies. †

Study	Subgroup	N =	OR(95% CI)	Hypertension definition	Exposure estimation	Age	Age breakdown
Our study		24238	1.06 (1.00-1.13)	Self-report	GIS	18-80	Yes
Bluhm et al 2007		667	1.90 (1.12-3.20)*	Self report	Manually	19-80	No
Järup et al 2008		4861	1.10(1.00-1.20)	Measurement	GIS	45-70	No
de Kluizenaar et al 2008	Groeningen	38849	1.03 (0.96-1.11)	Self-report	GIS	28-75	Yes
	PREVEND	7264	1.08 (0.95-1.23)	Measurement	GIS	28-75	Yes

Key characteristics, odds ratios (OR) and 95% confidence intervals (CI) are given.

* recalculated from 5dB increase

† Articles published after review by Babisch in 2006. Pubmed search "road traffic noise hypertension" years: 2007- 2009. Articles that did not present a continuous analysis were excluded.

A Swedish report found that the effect of road traffic noise on hypertension was stronger among study subjects that had lived in the same residence for more than 10 years[16] However, this study only adjusted for age and not for effect modification by age and the result is therefore hard to interpret. No effect modification by years in residence per se was detectable in our study, which could be explained by the fact that most subjects had lived in their current residence for several years, thereby limiting the misclassification of long-term exposure.

Although a strong association between road traffic noise and hypertension have been reported among females compared to males,[14] results are far from consistent[13,15] Large differences in effect between males and females could be ruled out with high statistical precision in our study. The effect did not vary markedly with respect to country of birth, strained economy or disturbed sleep but the statistical precision was much lower for these subgroups.

The hypertension prevalence in this study, as in other studies based on self-reports, are most likely substantially underestimated. However, if the misclassification of hypertension is non-differential with respect to road noise exposure results are biased towards the null.

Findings suggesting differences in effect across age groups may have several possible explanations. One explanation for the absence of effect among the elderly could be that the effect of noise may become less important, or harder to detect, relative to other risk factors with increasing age. Another explanation could be that noise annoyance varies with age. A recent meta-analysis showed that the association between age and noise annoyance was bell-shaped[30] Our finding can be interpreted as support for the suggested causal relation between annoyance and hypertension [31] though it recently has been questioned by a Norwegian study which, however, used age as a binary variable (over/under 70 years). [32] Earlier onset of disease rather than increased life-time risk is another possible explanation yet to be explored.

Age and years in residence are interrelated and separating the modifying effects can therefore be problematic. Stratifying the effect estimate by years in residence may reduce misclassification of long-term exposure in studies where exposure is assessed only for the current residence.

## Conclusion

The evidence for an association between transportation noise and cardiovascular risk in general is increasing[7] In our study, the effect of road traffic noise was only marked at high average levels (> 60 dB(A)), levels that were rare in our target population of southern Sweden. Road traffic noise may therefore not be as important determinant of cardiovascular risk in our target population as in other, noisier, urban environments. Impact on public health can be substantial in such environments, even if the effect is modest and restricted to middle-aged. Studies that have access to data on objectively assessed hypertension and residential histories should be encouraged. We also recommend that future studies use separate relative effect models for different age groups.

## Abbreviations

BMI: Body mass index (weight in kg divided by height in meters squared); GIS: Geographical information system; PM10: Particles with aerodynamic diameter less than 10 micrometers; dB(A): A-weighted sound level in decibel; LAeq24 h: day-night average noise level; OR: Odds ratio; 95% CI: 95% confidence interval.

## Competing interests

The authors declare that they have no competing interests.

## Authors' contributions

All authors of this paper have read and approved the final version submitted. They have also directly participated either in the planning, execution, or analysis of this study. I, TB did the statistical analysis and drafted the paper. MA developed the study design together with JB who also supervised the study. ES and JA did the GIS modeling and wrote parts of the paper's method section. P-OÖ chaired the health survey project that supplied much of the data. All authors have revised drafts and contributed to the discussion.
